# The Status of Irritability in Psychiatry: A Conceptual and Quantitative Review

**DOI:** 10.1016/j.jaac.2016.04.014

**Published:** 2016-07

**Authors:** Pablo Vidal-Ribas, Melissa A. Brotman, Isabel Valdivieso, Ellen Leibenluft, Argyris Stringaris

**Affiliations:** a Institute of Psychiatry, Psychology and Neuroscience, King’s College London, United Kingdom; b Section on Bipolar Spectrum Disorders, Emotion and Development Branch, National Institute of Mental Health, National Institutes of Health, Department of Health and Human Services, Bethesda, MD

**Keywords:** irritability, depression, anxiety, conduct, meta-analysis

## Abstract

**Objective:**

Research and clinical interest in irritability have been on the rise in recent years. Yet several questions remain about the status of irritability in psychiatry, including whether irritability can be differentiated from other symptoms, whether it forms a distinct disorder, and whether it is a meaningful predictor of clinical outcomes. In this article, we try to answer these questions by reviewing the evidence on how reliably irritability can be measured and its validity.

**Method:**

We combine a narrative and systematic review and meta-analysis of studies. For the systematic review and meta-analysis, we searched studies in PubMed and Web of Science based on preselected criteria. A total of 163 articles were reviewed, and 24 were included.

**Results:**

We found that irritability forms a distinct dimension with substantial stability across time, and that it is specifically associated with depression and anxiety in longitudinal studies. Evidence from genetic studies reveals that irritability is moderately heritable, and its overlap with depression is explained mainly by genetic factors. Behavioral and neuroimaging studies show that youth with persistent irritability exhibit altered activations in the amygdala, striatum, and frontal regions compared with age-matched healthy volunteers. Most knowledge about the treatment of irritability is based on effects of treatment on related conditions or post hoc analyses of trial data.

**Conclusion:**

We identify a number of research priorities including innovative experimental designs and priorities for treatment studies, and conclude with recommendations for the assessment of irritability for researchers and clinicians.

Irritability describes proneness to anger.^1^ As many other psychiatric concepts, before it entered nosology, it was the domain of poets,^2^, ^3^ philosophers,^4^, ^5^ and theologians.^6^ It was featured in Burton’s concept of melancholia,^7^ and it was termed “krankhafte Reizbarkeit” (literally, pathological irritability) in Bleuler’s textbook of psychiatry.^8^ It also has a venerable history in psychoanalysis, self-directed hostility underlying what Freud described as “the undoubtedly pleasurable self-torture of melancholy.”^9^

Irritability was omnipresent in all recent versions of the *DSM* as a symptom of psychiatric disorders and is, alongside lack of concentration and restlessness, one of the few symptoms to cut across externalizing and internalizing disorders.^10^ Chronic severe irritability as the primary feature of a new diagnostic category was introduced in the *DSM-5* in response to the controversy regarding the debate over the diagnosis of bipolar in children.^11^, ^12^ Over the last 10 to 15 years, the number of prepubertal children diagnosed with bipolar disorder (BD) in the United States has increased at rates close to 500%.^13^, ^14^ This increase was thought to result partly from counting severe and chronic irritability of early onset (“present forever” or “since the first year of life”) as a cardinal manic symptom, analogous to the classical cardinal manic symptoms of elated mood or episodic irritability.^12^ The diagnosis of disruptive mood dysregulation disorder (DMDD)^10^ was an attempt to curb what was seen as an overdiagnosis of BD, while recognizing the burden of problems suffered by children whose primary problem was chronic severe irritability, for whom there was no diagnostic home in the *DSM*.

In the Research Domain Criteria (RDoC) framework,^15^ irritability is regarded as an expression of frustrative nonreward, which is defined as the reaction to blocked goal attainment. Conceptualizing irritability as a response to blocked goal attainment allows for research across species. Moreover, seeing irritability as a dimension is in line with a broader recognition within psychiatry that phenotypes may lie on a quantitative spectrum rather than being discrete categories.^16^

The introduction into *DSM* of chronic severe irritability as a nosological category of its own has led to inevitable questions about its conceptual foundations as well as about its reliability and validity. In this review, we set out to answer those questions. We start by providing a conceptual background about irritability, offering a working definition and delineating its relationship to key concepts such as reward, emotion, mood, aggression, and normal variations in behavior. We then set out to address questions about reliability and validity. We do so guided by both the Robins-Guze criteria^17^ and the Cronbach and Mehl validity considerations.^18^ We first examine whether irritability forms a statistically distinct factor, before looking at how reliably it can be measured across time and informants. After that, we present the results of a systematic review and meta-analysis about the longitudinal predictions of irritability. We then turn to its etiological underpinnings by examining the available genetic and neuroimaging literature. We conclude with a discussion of our findings and delineate several research priorities.

## Working Definition and Conceptual Background

Irritability refers to interindividual differences in proneness to anger that may reach a pathological extent. We deliberately use this broad definition as it allows us to discuss the boundaries of irritability, for example, issues about whether such reactions are normative (see definition published elsewhere^19^) or whether a behavioral component such as aggression is required (see elsewhere, for example^20^). In keeping with Karl Popper, the philosopher of science, we see the role of a definition as “cutting a long story short” rather than as condensing all knowledge on the subject. As in all other areas of science, the defining terms are in themselves hard to define—neither “proneness” nor “anger” are semantically unequivocal. Below we explore such terms related to irritability in an attempt to prevent what has been described as “quarrelling about words.”^21^

### Feelings, Emotions, Mood, Affect, and Irritability

It is useful both clinically and for the design of future experimental studies to discuss the relationship between irritability and the key concepts of feeling, emotion, mood, and affect.

Anger, the defining feature of irritability, can be conceptualized as a feeling but also as an emotion. Anger is a feeling in that individuals are consciously aware of a set of thoughts and bodily sensations that they describe as anger. Similarly, an individual may sense a proneness to anger as feeling on edge or feeling touchy—another description for feeling irritable. On the other hand, emotions are thought of as action tendencies that do not need to enter conscious awareness, although they may do so. Anger can be an emotion in that people may act in an angry way without being aware of it (i.e., without feeling the anger). Experimentally, this is demonstrated through the subliminal presentation of stimuli and assessment of the participants’ response biases.^22^ The concept of mood is used by clinicians to describe states that are valenced (i.e., negative as in depression or positive as in mania) and enduring. How enduring such a state has to be in order to be called a mood rather than an emotion is, however, unclear. Also, although emotions are evoked responses to stimuli, the definition of mood does not entail a stimulus. Irritability is a mood in the sense that young people can remain in states of proneness to anger for very long times and sometimes for no apparent reason, as discussed below. Typically psychiatrists assess mood by accessing their patient’s feelings, although clinicians will also resort to descriptions of patients by others, such as when asking parents to rate their child’s irritability. Finally, in psychiatry, the term “affect” describes the observed features of a mood or emotion, such as angry facial expression in someone who is irritable during interview.

In summary, irritability is a mood, and anger is its defining emotion. When anger enters the person’s awareness, it is called a feeling, and when observable to others, such as clinicians, anger is described as an affect. The reader should interpret these statements cautiously because the boundaries between the concepts of mood and emotion are not firmly grounded in empirical data and because in a lot of the literature, mood and emotion are used synonymously.

### The Phasic and Tonic Components of Irritability

An alternative clinical conceptualization of irritability is presented in the *DSM-5* and derives from the work of Leibenluft *et al.* defining the syndrome of severe mood dysregulation (SMD), the precursor to DMDD.^12^, ^23^ The *DSM-5* criteria for DMDD identify 2 components of irritability. Phasic irritability refers to developmentally inappropriate temper outbursts, whereas tonic irritability refers to the negative affect that persists between outbursts. These 2 components were included in SMD because the temper outbursts are the hallmark clinical feature of these children, whereas requiring the tonic component allowed the investigators to recruit a sample of children who, like those with BD, have a severe and persistent mood disorder. The latter was important because SMD was operationalized to compare youth with chronic, persistent, severe irritability (i.e., the SMD phenotype) to youth who have distinct manic episodes characterized by elated mood (i.e., classic BD). However, it is unclear whether tonic and phasic irritability are distinct constructs in terms of validators such as longitudinal clinical predictions, treatment response, pathophysiology, or family history. The 2 constructs are difficult to distinguish in secondary analyses of epidemiological data,^24^ yet the distinction needs to be tested in clinical data using instruments specifically designed to capture each component. The *DSM-5* account of irritability has the advantage of being purely descriptive and not having to resort to concepts such as emotion or mood that are hard to define in their own right.

### Approach Tendencies, Reward, and Irritability

**Figure 1:**
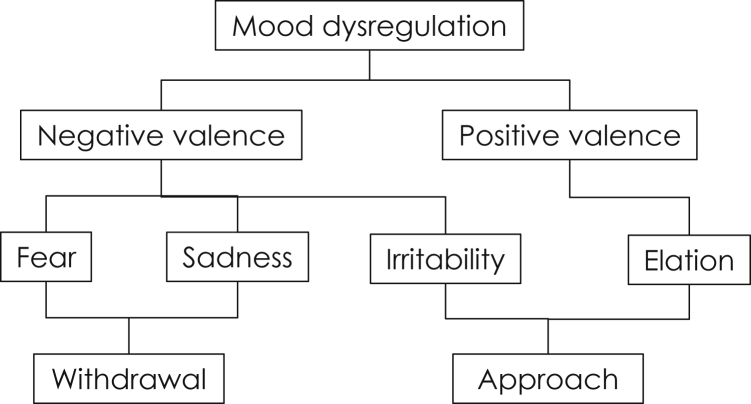
The position of irritability within commonly used terminology. Note: irritability shares a negative valence with anxiety and depression but denotes approach and is therefore linked to elation in mania.

Irritability and its defining feeling of anger are classified as negatively valenced and therefore, by definition, as unpleasant, as with fear or sadness. However, although people typically avoid fear-inducing stimuli, people will approach anger-inducing stimuli in experimental paradigms.^22^ Moreover, people who score high on angry traits are also more likely to score highly on behavioral approach tendencies and the seeking of rewards.^25^ This unique position of irritability among emotional valence and approach/avoidance tendencies, as described by Stringaris and Taylor,^1^ is depicted in Figure 1. Indeed, irritability has been linked with the frustration that arises when a goal is blocked.^26^ Such a goal may be strictly defined (e.g., being prevented from obtaining a promised monetary reward) or may be more complex and embedded within social relationships (e.g., experiencing an insult or similar social setback). The clinical equivalent of what experimental psychologists describe as an approach tendency may be the propensity to fight (literally or metaphorically) to obtain a blocked response. In such situations, irritability has 2 effects. First, it reflects the motivation behind continued attempts to obtain the reward. Second, a child’s irritability can evoke negative feelings in parents or peers—these feelings may range from fear and avoidance (or accommodation in the case of the parents) to mutual feelings of anger. In that sense, irritability can be a potent environment-modifying emotion. Relationships between parents and children that are characterized by irritability have been described as mutually coercive^27^ and are the target of parenting interventions.

### Irritability and Aggression

Verbal or physical aggression is often the trigger for referring irritable youth to mental health services.^28^ Yet irritability is by no means the same thing as aggression. Shouting or fighting (parts of the definition of aggression) can arise out of escalated anger; however, irritable children may simply be grumpy, huffing and puffing, or experiencing burdensome dysphoria (“stewing inside”), rather than exhibiting aggression. Separating irritability from aggression is therefore important for 2 reasons: first, because recognizing it might prevent the manifestation of aggression; second, because irritability may be burdensome independent of aggressive acts.

### Boundaries to Normality and Dysregulation

Anger can be a normal reaction^1^ and can vary along with other temperamental traits^29^; it can also predict significant impairment throughout life.^19^, ^30^ Deciding where to set the threshold above which irritability would be considered pathological can be difficult and may vary, depending on factors such as the young person’s age or environment. Because clinicians must make binary decisions (“to treat or not to treat”), having a threshold is important. However, such cut-offs are often arbitrary, and, as we shall see below, this arbitrariness may have a negative impact upon reliability and validity. It is possible to derive empirical thresholds, for example, defining levels of irritability based on the amount of associated impairment.

The term “dysregulation”—frequently used to describe irritability (as, for example, in DMDD)—entails a judgment about what is pathological. Irritability is one of many manifestations of mood dysregulation.

With these discussions about normativity in mind, it is not surprising that estimates of the prevalence of irritability can vary substantially. For example, lifetime prevalence of SMD was observed to be about 3.3% in children and adolescents aged 9 to 19 years.^31^ The rates of DMDD were similar (3.3%) in a sample of preschool children.^32^ However, these rates decreased to 1% in 2 samples of older youth.^32^ Studies examining mood dysregulation and mood lability, which includes irritability as 1 of its manifestations, have reported higher prevalence estimates. In community studies, 5% to 6% of youth aged 8 to 19 had a lot of mood lability,^33^ and 3.8% of youth aged 4 to 16 years presented with the Child Behavior Checklist (CBCL) Dysregulation phenotype,^34^ which is linked to irritability. Finally, about 20% of adolescents participating in the Isle of Wight study^35^ were rated as displaying significant irritability in terms of frequency, severity, and duration.

## Multivariate

*Specific Question*: Does irritability form a factor that can be distinguished from other psychiatric constructs in multivariate statistical analysis?

The application of factor analysis as a method of construct validation has a long history in the development of psychological constructs^18^ and has also been used to validate irritability.

Irritability, operationalized as touchiness, easy annoyance, and anger, was shown to have differential predictions compared to other symptoms of oppositional defiant disorder (ODD), such as defiance or vindictiveness.^36^ This finding prompted a series of either exploratory factor analyses (where no specific hypothesis is being tested),^37^ or confirmatory factor analyses.^37^, ^38^, ^39^, ^40^, ^41^ These published analyses demonstrate that irritability is a distinguishable dimension within ODD. Importantly, some of these articles have shown the superiority of considering irritability^41^ as an independent dimension over several other alternative models.^42^, ^43^ A recent comprehensive analysis of 5 different samples compared a variety of models and concluded that an irritable dimension defined as anger, temper outburst, and easy annoyance^36^ fit the data best.^39^

The independence of irritability as a construct has also been confirmed using latent class analysis.^40^, ^44^, ^45^, ^46^ Unlike factor analysis, which examines dimensions within the data, this method aims to identify groups of people based on their response to a questionnaire, for example.^47^ This method, therefore, is closer to the binary constructs that clinicians use.

*Conclusion*: In both factor and latent class analyses, irritability can be differentiated from other symptoms of oppositional behavior; however, its distinctiveness from other items of psychopathology has yet to be demonstrated.

## Reliability

*Specific Question:* Can irritability be measured reliably?

The word “reliable” here refers to whether a measure yields consistent results across different conditions or time points.^18^ Height measurements are a prototype of reliable measurements of physical health, and IQ measurements are also consistent, at least after a certain age.^48^ Reliable measurement of a psychological construct, such as irritability, is a prerequisite for testing its validity (i.e., testing whether the measure captures what it is purporting to be measuring). The reliability of irritability has been assessed in the following ways:

#### Internal Consistency

This assesses how well items of a scale correlate with each other. The average internal consistency of irritability scales is high (α = 0.75),^19^, ^37^, ^40^, ^49^, ^50^, ^51^, ^52^, ^53^ ranging from 0.49 (for ad hoc*−*created scales)^19^ to 0.92 (for specifically developed irritability scales).^50^

#### Test−Retest Reliability (or Longitudinal Stability)

The goodness of test*−*retest reliability differs according to whether irritability is measured continuously or categorically. Studies using dimensional approaches show that irritability is moderately stable over time, with correlations ranging between 0.29 and 0.88.^50^, ^52^, ^53^, ^54^ Recently, a study of 2,620 children aged 8 to 9 years followed up for more than 10 years found that both parent- and self-reports of irritability were moderately correlated over time (r parent, 0.32–0.49; r self, 0.31–0.45).^55^

However, as compared with other psychiatric disorders, the current categorical definition of irritability shows low stability across time. *DSM-5* field trials have shown poor test*−*retest reliability for DMDD (κ = 0.25; 95% CI = 0.15−0.36).^56^ In a clinical sample of children with DMDD aged 6 to 12 years, only 19% met criteria at 1- and 2-year follow-up.^57^ Similarly, findings over 4 time points in the Great Smoky Mountain study showed that most youth with SMD (82.5%) met SMD criteria in 1 wave, but only 1.4% met criteria in all 4 waves of assessment.^31^ However, findings on the same sample showed that youth with either persistent anger or temper outburst had a 75% likelihood of having either persistent anger or temper outburst 1 year later.^24^

Thus, it appears that irritability defined stringently and categorically remains chronic only in a small proportion of children. The lack of stability of categorically defined irritability is probably because a particular, artificially set threshold is not met: for example, thresholds that stipulate the frequency of temper outbursts or duration of negative mood. Therefore, a high proportion of children who do not meet current DMDD criteria still present with impairing chronic irritability.^58^ Recently, a study evaluated only the main symptoms of DMDD (i.e., irritable/angry mood and temper outburst) in a community sample of children followed up for more than 8 years.^59^ The authors found that although rates of symptom remission were high (71%), the prevalence of new cases was also considerable (55%). Moreover, 29% of the participants with frequent DMDD symptoms at baseline also displayed these symptoms at follow-up.^59^

#### Interrater Reliability

In a German sample of 1,031 children and adolescents aged 10 to 18 years,^38^ the correlation between parent and child reports of irritability was r = 0.32, very similar to the results found in a recent longitudinal study (r = 0.23−0.36) with 2,620 Swedish children.^55^ These 2 studies used items from the CBCL to measure irritability. A study using a specific measure of irritability (i.e., the Affective Reactivity Index) found that parent- and self-report scales were strongly correlated in both a US sample (r = 0.58; 95% CI = 0.47–0.66) and a UK sample (r = 0.73; 95% CI = 0.56–0.85).^50^ However, in other samples (Dr. Joel Stoddard, NIMH, personal communication), correlations were within the bounds of what one would expect according to a meta-analysis on cross-informant agreement in behavioral and emotional problems.^60^

*Conclusion*: Irritability shows mostly good internal consistency and substantial longitudinal stability when measured continuously. Its test−retest reliability as the categorical construct of DMDD is poor, probably because children may fall just below threshold but still be impaired by irritability.^58^ Interrater reliability of irritability, specifically between self- and parent reports,^60^ is consistent with the reliability of other behavioral and emotional constructs.

## Longitudinal Outcomes

*Specific Question*: Does irritability have longitudinal predictions that differentiate it from other disorders?

A key question is what happens to irritable children over time. Are interindividual differences in irritability innocuous, or are they useful in predicting trajectories of future impairment? Also, is irritability an antecedent of specific future problems, or rather a predictor of a broad array of difficulties? To answer these questions, we use a quantitative approach, that is, a meta-analysis. We also provide data on future functional outcomes associated with irritability.

In Supplement 1, available online, we describe in detail the methods used for the meta-analysis, including data sources, search strategies, inclusion/exclusion criteria, study selection, data extraction, and data analysis. Additional analytic steps, such as removing separate studies conducted on the same sample or attempts to explain heterogeneity and publication bias, are presented in the main text only if they influence the main conclusions; otherwise, they are presented in the supplemental material.

Briefly, we meta-analyzed longitudinal studies in which chronic nonepisodic irritability was the predictor of future psychiatric disorders, as these are most clinically relevant. We conducted the meta-analysis for findings where outcome data from 2 or more studies of different cohorts could be combined, and then calculated pooled odds ratios (ORs) and 95% CIs for each possible outcome. We also reviewed longitudinal studies predicting continuous outcomes (i.e., symptom scores) or providing descriptive statistics. When possible, these studies were subjected to a separate meta-analysis, and pooled standardized β coefficients and 95% CIs were calculated. Otherwise, only a description of the findings is provided.

When 2 or more studies from the same cohort predicted the same outcome, each of these studies was individually excluded from the analysis, and the pooled estimates and 95% CIs were recalculated. If an individual study from a specific cohort contributed heavily to the pooled estimate, a change in the magnitude or significance of the pooled estimate would be observed.

We tested for between-study heterogeneity using the *I*^2^ statistic, which is the percentage of variation attributable to heterogeneity. The values of *I*^2^ lie between 0% and 100%, with larger values showing increasing heterogeneity. Higgins *et al.*^61^ suggest that *I*^2^ values between 25% and 50% are low, between 50% and 75% moderate, and for ≥75% high.

Finally, given the possibility of publication bias (i.e., significant findings being more likely to be published), we examined whether there was asymmetry in funnel plots (a scatterplot of the estimates from individual studies against a measure of a study size) and calculated the Egger’s coefficient bias.^62^

### Characteristics of Studies for the Meta-Analysis

The search and review of studies resulted in 12 studies appropriate for inclusion in the meta-analysis for the prediction of psychiatric disorders,^19^, ^30^, ^31^, ^35^, ^43^, ^44^, ^49^, ^57^, ^63^, ^64^, ^65^, ^66^ and 12 studies predicting continuous outcomes^37^, ^40^, ^42^, ^46^, ^52^, ^53^, ^67^, ^68^, ^69^, ^70^ or appropriate for descriptive analysis^58^, ^71^ (see Figure S1, available online). In addition, 5 studies among those included in the meta-analysis provided information about future functional outcomes,^19^, ^30^, ^35^, ^49^, ^63^ including suicidal behaviors.

Information about the 12 studies included in the meta-analysis for the prediction of psychiatric disorders is presented in Table S1, available online. These studies comprised 9 cohorts and 7,594 unique participants. The analyses included the prediction of depression, anxiety disorder, BD, attention-deficit/hyperactivity disorder (ADHD), ODD, conduct disorder (CD; antisocial personality disorder in adulthood), and substance disorder.

Below we present the results of the meta-analysis for the prediction of each disorder. Studies predicting continuous outcomes are analyzed and described in detail in the supplemental material, available online, and only the main results of those studies are described here. Results from studies predicting functional impairment are described in a separate section at the end.

### Chronic Irritability as a Predictor of Psychiatric Disorders

**Figure 2:**
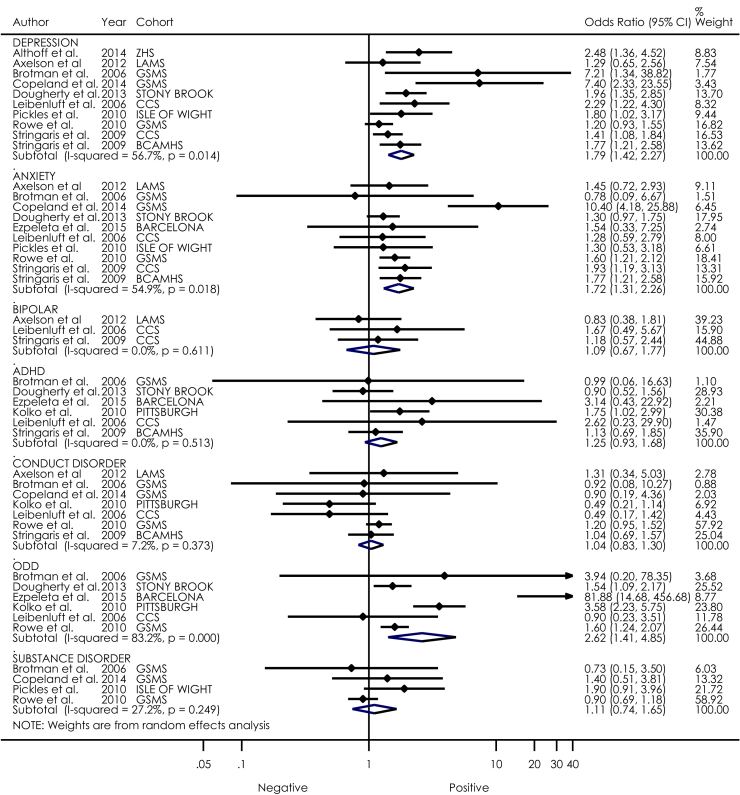
Forest plot of irritability as a predictor of future psychiatric disorders. Note: Points represent the estimated odds ratio of each study; the lines bisecting the point correspond to the 95% CI. Pooled effect sizes are represented by diamonds. Weights for each study are given in the far-right column. ADHD = attention-deficit/hyperactivity disorder; ODD = oppositional defiant disorder.

Figure 2 shows effect sizes and the corresponding 95% CIs for each study in the prediction of future psychiatric disorders from irritability. Results are presented for each psychiatric disorder separately.

#### Depressive Disorder

Ten studies representing 7 cohorts included depressive disorder as an outcome. Of those 10 studies, 8 reported significant findings, with irritability predicting depression at follow-up (OR = 1.80, 95% CI = 1.42−2.27, *p* < 0.001). The overall variance of these results was moderate (*I*^2^ = 56.7%), suggesting that 57% of heterogeneity was due to covariates.

#### Anxiety Disorders

Ten studies representing 7 cohorts included anxiety disorder as an outcome. Irritability was a significant predictor of anxiety at follow-up (OR = 1.72, 95% CI = 1.31−2.26, *p* ≤ .001). The overall variance of these results was also moderate (*I*^2^ = 54.9).

#### Bipolar Disorder

Three studies representing 2 cohorts included BD as the outcome. No study reported significant findings. When all of these studies were considered together, chronic irritability was not a significant predictor of BD at follow-up (OR = 1.09, 95% CI = 0.67−1.77, *p* = .739). The overall variance of these results was low (*I*^2^ = 0%).

#### Conduct Disorder

Seven studies representing 5 cohorts included CD as an outcome. Of those, no study reported significant findings. When all of these studies were considered together, irritability was not a significant predictor of CD at follow-up (OR = 1.04, 95% CI = 0.83−1.30, *p* = .735). The overall variance of these results was low (*I*^2^ = 7.2%).

#### ADHD

Six studies representing 6 different cohorts included ADHD as outcome. Of those, only 1 study reported significant findings.^64^ When all the studies were considered together, irritability was not a significant predictor of ADHD at follow-up (OR = 1.25, 95% CI = 0.93−1.68, *p* = .139). The overall variance of these results was low (*I*^2^ = 0%).

#### ODD

Six studies representing 5 cohorts included ODD as an outcome. When all of the studies were considered together, irritability was a significant predictor of ODD at follow-up (OR = 2.62, 95% CI = 1.41−4.85, *p* = .002). However, the overall variance of these results was high (*I*^2^ = 83.2). It should be noted that this association is very likely to have been inflated by item overlap.

#### Substance Abuse/Dependence

Four studies representing 2 cohorts included substance disorder as an outcome. Of those, no study reported significant findings. When all of the studies were considered together, irritability was not a significant predictor of drug abuse at follow-up (OR = 1.11, 95% CI = 0.74−1.65, *p* = .613). The overall variance of these results was low (*I*^2^ = 27.2%).

Test of publication bias was examined only for the prediction of depression and anxiety, as these were the only outcomes with 10 studies, which is the minimum number of studies recommended for test of publication bias.^72^ For anxiety, there was no evidence of publication bias. For depression, the Egger bias coefficient suggested the presence of asymmetry and publication bias (bias = 2.60, *p* = .004) toward positive results, but this asymmetry was accounted for by 2 studies^30^, ^31^ and disappeared when these 2 studies were removed from the analyses (OR = 1.61, 95% CI = 1.35−1.93, *p* = .076, *I*^2^ = 34.6%; bias = 2.21, *p* = .067) (see Figures S2 and S3, available online). Most of the remaining individual effect estimates were greater than 1; therefore, the effect of publication bias, if any, would be to inflate the estimate rather than to lead to an incorrect conclusion about the existence of an effect. Test of publication bias in the prediction of the remaining outcomes (although these included fewer than 10 studies) yielded nonsignificant results. A detailed description of examination of publication bias is provided in the supplemental material, available online.

### Prediction of Continuous Outcomes

Information about the 12 studies predicting continuous outcomes or appropriate for descriptive review is presented in Table S2, available online. Consistent with the results for categorical outcomes, studies using continuous outcomes (i.e., symptom scores) found that irritability is associated with future depression^37^, ^42^, ^46^, ^52^, ^67^, ^68^, ^70^ and internalizing symptoms (i.e. depression and anxiety together),^40^, ^53^, ^64^, ^69^ with pooled effect sizes (ES) ranging from 0.12 to 0.21 (*p* < .001) (see supplemental material, available online).

### Prediction of Functional Outcomes

Irritability in youth has been associated with lower financial and educational attainment^19^, ^30^ as well as worse health outcomes in adulthood.^30^ The association between irritability and future functional impairment has also been found in preschool children.^49^, ^63^ Finally, 1 study found that irritability in adolescence was associated with suicidal behaviors in adulthood independent of affective diagnoses (OR = 3.2; 95% CI = 1.9–5.3; *p* < .001).^35^

Exploratory analyses of covariates that could explain the variability of effect sizes for the prediction of depression, anxiety, and ODD were not significant. A detailed description of these analyses can be found in the supplemental material, available online.

*Conclusion*: Irritability is associated specifically with future depression and anxiety problems. In addition, irritability appears to be associated with future impairment, even when adjusting for baseline psychopathology.

## Etiological Underpinnings

*Specific Question*: Comparing irritability with other psychiatric traits, what shared and distinct pathophysiological mechanisms can be discerned?

At least since the work of Robins and Guze,^17^ attempts have been made to validate psychiatric disorders by showing distinct pathophysiology, such as genetics or brain physiology. Thus far, this has proved difficult to achieve for common psychiatric disorders, such as depression, anxiety, or schizophrenia. Irritability—defined either as a trait, as SMD or as DMDD—is no exception. In this section, we present evidence from genetics and neuroimaging, mainly because the vast majority of etiological studies come under 1 of these headings.

### Genetically Informative and Family Studies

Twin studies seek to estimate the heritability of a trait (i.e., irritability) by comparing the phenotypic (e.g., behavioral, emotional) similarity of monozygotic twins, who are genetically identical, with that of dizygotic twins, who share on average 50% of their genes. These studies also allow us to decompose the observed variance of a trait into its genetic, shared environmental, and nonshared environmental components. Shared environmental factors are those environmental influences that cause siblings in the same family to be similar to one another (e.g., growing up in the same neighborhood, the family’s socioeconomic status), whereas nonshared environment factors are environmental influences that cause siblings in the same family to be different from one another (e.g., having different friends, experiencing different life events).^73^

#### Heritability

The genetic contribution to the variation of irritability is approximately 30% to 40% in both adults^74^ and adolescents.^37^ This is close to the heritability estimates for depression and anxiety.^75^ Genetic influences on irritability increase slightly over time in males and decrease in females,^55^ and unique (as opposed to shared) environmental factors explain most of the remaining (non-genetic) variance in both.

#### Family History

A family history of depression has been associated with the ODD irritability dimension,^41^ and maternal depression predicts irritability in young children.^76^ In addition, the relation between maternal history of depression and adolescent depression is mediated partly by the presence of irritability in childhood.^70^ Although one study showed no differences in parental history of psychiatric disorders between youth with and without DMDD,^57^ parents of youth with narrow BD were more likely to be diagnosed with BD than parents of youth with SMD.^77^

#### Overlap Between Irritability and Depression

A twin genetic study showed that irritability and depression share substantial genetic variance,^37^ that is, the longitudinal association between irritability and depression is, to a significant extent, due to overlapping genetic factors. By contrast, irritability and depression differ phenotypically because of unique environmental factors. This finding is in keeping with what is known from other psychiatric phenotypes, such as depression and anxiety.^78^

Recently, a longitudinal study found that genetic covariance between irritability and anxiety/depression is highest in early adolescence (74%), and that the impact of irritability on future internalizing symptoms is higher than the impact of internalizing symptoms on future irritability.^69^

### Neurocognitive Mechanisms

Research on behavioral and neurobiological mechanisms in irritability has recently started to emerge. To date, most of this research has focused on differentiating SMD from BD and healthy volunteers (HV)^20^ and, to lesser extent, from ADHD^79^, ^80^ and depression/anxiety.^80^ Here, we present the main findings in behavioral experiments and structural and functional imaging studies.

#### Behavioral Results

Three main behavioral findings have been reported in youth with SMD. First, young people with chronic severe irritability show face emotion labeling deficits across emotions.^80^, ^81^, ^82^ This abnormality is not unique to children with irritability but is also seen in youth with BD^83^ and other psychopathology.^84^ Second, youth with SMD show aberrant face emotion threat processing, as evidenced by showing attentional bias toward threatening faces^85^ and by perceiving neutral faces as more threatening^79^ than do HV. Bias toward threatening faces has also been found in irritable children from a community sample (unpublished data, October 2015). In addition, a recent study found that youth with DMDD rated ambiguous faces as more angry than did HV.^86^ The bias toward threatening faces is not unique to SMD but is also present in people with depression and anxiety.^87^, ^88^

Finally, compared with HV, youth with SMD have deficits in reversal learning,^89^ performing even worse than youth with BD when attentional demands increase.^89^

#### Structural Neuroimaging

To our knowledge, there is only one study on structural magnetic resonance imaging (MRI) examining differences among youth with SMD, youth with BD, and HV.^90^ Cross-sectional findings revealed that, compared with HV, youth with SMD and BD had greater gray matter volume in the presupplementary motor area, dorsolateral prefrontal cortex, and insula. However, abnormally increased parietal and precuneus volume was seen only in participants with BD,^90^ suggesting that SMD and BD may differ in structural brain development.

#### Functional Neuroimaging

Overall, two main deficits have been associated with SMD: namely, alterations in the processing of emotional stimuli, and alterations in adapting to changing environments.

#### Processing of Emotional Stimuli

Functional MRI (fMRI) paradigms examining face emotion processing can probe conscious processing of face emotions (i.e., unmasked faces presented for ≥40 milliseconds) or nonconscious (i.e., masked faces presented for <40 milliseconds). Unmasked processing can be implicit, in which research participants focus on a stimulus feature other than the face emotion (e.g., reporting nose width or gender), or explicit, in which the task directs attention toward the face emotion (e.g., rating fear or hostility). Finally, there are also affective priming paradigms, in which the response to a neutral stimulus is influenced by the previous presentation (either masked or unmasked) of an emotional face.

To date, research findings suggest that youth with SMD display amygdala dysfunction. Early evidence suggests that amygdala hypoactivity may be present during explicit processing of face emotions,^79^ while amygadala hyperactivity is evident during implicit processing, compared to that in HV^79^, ^91^ as well as to youth with BD or ADHD.^79^ In addition, 1 study combining explicit and implicit processing found inappropriate modulation of amygdala activity with increasing anger in faces in youth with SMD compared with HV,^92^ again supporting an amygdala dysfunction hypothesis in SMD.

Two studies using affective priming tasks with both masked and unmasked emotional faces^93^, ^94^ found that youth with SMD show higher activation during viewing of angry faces in the posterior cingulate and superior temporal gyrus compared to HV.^93^, ^94^ One of these studies also found hyperactivation in the insula, parahippocampal gyrus, and thalamus in the same contrast, and the same regions showed hypoactivation when processing happy faces.^94^ In contrast, compared to BD, youth with SMD show lower activity in parietal, temporal, and frontal regions when processing neutral faces.^93^

Aberrant response to emotional stimuli in the amygdala and frontal regions is consistent with recent findings in functional connectivity during resting state fMRI.^95^ In this study, youth with BD showed higher functional connectivity between the left basolateral amygdala and the medial superior gyrus and posterior cingulate than participants with SMD.^95^

#### Ability to Adapt to Changing Environments

The capacity to adapt behavior in response to changes in environmental contingencies (e.g., when progress toward a goal is blocked) is called context-dependent regulation. In experimental settings, 2 paradigms have been used to assess this adaptive ability: reversal learning paradigms, in which the rewarded object (A versus B) changes continuously and the individual must detect the change in reward contingencies; and frustration paradigms, in which frustration is induced by changing reward contingencies so that participants are unable to attain a desired reward.

During a response reversal task, the difference in activation between correct and incorrect trials was smaller in youth with SMD compared to HV in the caudate, and smaller in the inferior frontal gyrus compared to both HV and BD.^96^ These regions are involved in learning from error signals and response inhibition, respectively, suggesting that youth with SMD have difficulties learning from errors and changing their behaviors accordingly. Differences between youth with SMD and BD were also shown in motor inhibition tasks, in which participants with BD showed less activation than youth with SMD and HV in the anterior cingulate cortex (ACC) and nucleus accumbens (NAcc) during failed inhibition.^97^

Few studies have used frustration paradigms in youth with severe irritability. These have shown that, under frustrating conditions, youth with SMD display aberrant amygdala, striatal, parietal, and posterior cingulate activations compared with HV,^98^, ^99^ suggesting difficulties in emotion regulation, reward processing, and attentional control. Moreover, youth with SMD differ from those with BD in their event-related potentials (ERPs)^100^ and brain activation patterns^101^ during frustration. The pattern of ERPs following frustration suggested impaired early attentional processes in youth with SMD compared with HV and those with BD, with alterations in frontal and temporal regions.^100^ Aberrant frontal activations, including those in the ACC and medial frontal gyrus, were also found in youth with SMD in response to negative feedback compared with HV, whereas youth with BD presented with decreased activation in the superior frontal gyrus and insula.^101^

#### Correlations With Irritability

Reports of associations between brain activations and dimensionally measured irritability are scarce. In preschool children with severe chronic irritability, higher levels of parent-reported irritability were negatively associated with ACC activation^99^ and positively associated with dorsolateral prefrontal cortex (PFC) activation^102^ during a frustration task. By contrast, in youth with SMD, parent- or child-reported irritability was negatively correlated with activation in the right thalamus during processing of happy faces and positively correlated with activation in the left ventromedial PFC during nonaware perception of emotional faces.^94^

*Conclusion*: Irritability is moderately heritable. Genetic factors underlie the phenotypic overlap between irritability and depression—indicating the shared etiology between the 2 disorders—although it remains unclear which genes are involved. Thus, similar to other psychiatric disorders, what makes irritability unique from depression are environmental factors (although it is unclear what these factors are). By contrast, genetic factors seem to be pleiotropic and mediate the overlap between irritability and other traits, very much in keeping with the current understanding that psychiatric disorders are comorbid with each other due to shared genes.^103^

Youth with SMD share aberrant processing with BD but also differ in functional and structural MRI findings. Overall, youth with SMD compared to healthy volunteers show altered activations in amygdala (emotional processing), striatum (reward processing and error learning), and frontal regions (attention, inhibition/cognitive control, alternative response).

## Treatment Response

*Specific Questions*: What are the most effective treatments for irritability? Can treatment tell us anything about the pathophysiology of irritability? Are there treatments that are specific to irritability?

Drawing etiological inferences from treatment response (*ex juvantibus*) is common in other areas of medicine, such as infectious diseases.^104^ Yet, in psychiatry, many different disorders may respond to the same treatment—for example, antipsychotics are useful in both schizophrenia and BD, and antidepressants can be effective in both depression and anxiety disorders. This might also be the case with irritability, for which so far there is no specific treatment and which may respond to a range of treatments.

To our knowledge, there is only 1 published RCT specifically treating irritability,^105^ which found no benefit of lithium over placebo in children with SMD. This was another important step in differentiating chronic irritability from mania. The only other published pharmacological study to have irritability as a primary outcome is a non-RCT open-label trial that used low doses of risperidone in youth with SMD and showed significant reductions in irritability scores.^106^

Irritability may respond to stimulant medication, at least in the context of ADHD. Recently, post hoc analyses in the Multimodal Treatment Study of Children with ADHD (MTA Study) found that stimulant (ES = 0.63) or stimulant plus behavioral treatment (ES = 0.82) was better than behavioral treatment alone (ES = 0.42) in reducing irritability symptoms.^107^ However, the magnitude of the effect sizes for the irritability response to treatment was approximately half of that for ADHD symptoms.^108^ These recent findings are consistent with results from studies of a related phenotype, aggression (i.e., 2 meta-analyses show moderate to large ES for stimulant treatment of aggression in ADHD^109^, ^110^). In addition, 1 study^111^ found that stimulants along with behavioral therapy reduced externalizing symptoms in children with ADHD and SMD. Moreover, a recent meta-analysis found that emotional instability in ADHD benefited from stimulants.^112^ Finally, the mood stabilizer divalproex added to stimulant has shown to be effective in children who remain aggressive despite optimized treatment of ADHD symptoms.^113^ It remains unclear, however, whether stimulants treat irritability directly or by decreasing ADHD symptoms first.

There is indirect evidence from the adult literature that selective serotonin reuptake inhibitors (SSRIs) may be effective in the treatment of irritability, aggression, and explosive outbursts in the context of mood disorders (depression, dysthymic disorder, premenstrual syndrome, or anxiety)^114^, ^115^ and intermittent explosive disorder.^116^ However, none of these trials has targeted irritability in itself. Trials of SSRIs following a stimulant lead-in phase are ongoing at the National Institute of Mental Health (NIMH) and University of California, Los Angeles (UCLA), but no other research in young people has been published.

Antipsychotics may be effective in decreasing irritability and aggression in children with sub-average IQ^117^ and autism spectrum disorders (ASD).^118^, ^119^ However, the extent to which irritability in those trials (typically measured by observation using the Aberrant Behavior Checklist) corresponds to irritability as defined in most studies quoted in this article remains unclear.^120^

For psychological interventions, parent training may be effective in reducing irritability in children with ODD^121^ and ASD.^122^ In addition, individual cognitive-behavioral therapy (CBT) has demonstrated effectiveness in youth with excessive anger^123^ and Tourette syndrome.^124^ Individual CBT also reduced temper outbursts in a clinical sample of young people with OCD and depressive symptoms,^125^ but this was not a trial design. Group CBT has been shown to be effective in 1 trial of youth with ADHD plus SMD.^126^, ^127^

Recently, and based on the work of Penton-Voak *et al.*,^128^, ^129^ Stoddard *et al*.^86^ tested whether the judgment of ambiguous faces as angry could be altered in youth with DMDD, and whether this change might be associated with reduced irritability. Using an open active training, the authors showed that the bias was reduced, as well as levels of irritability, and that this reduction was associated with changes in neural activity in the orbitofrontal cortex (OFC) and amygdala.^86^

*Conclusion*: Very few trials have been conducted with irritability as a primary outcome. Most knowledge about the treatment of irritability is based on effects of treatment on related conditions (e.g., aggression or ODD), or through post hoc analyses of trial data (e.g., irritability in those with ADHD).

## Discussion

The main aim of this review was to assess the status of irritability in nosology by examining whether irritability is a reliable and valid construct. Using a combination of selective and systematic reviewing as well as a meta-analysis, we found that irritability can be measured reliably and that it forms a distinguishable factor with distinct longitudinal predictions. This is particularly evident for irritability as a dimensional construct, which shows reliability and predictive value that is comparable to that in other psychiatric entities, such as anxiety or hyperactivity. Moreover, we showed that irritability is a robust predictor of future emotional disorders, particularly depression, as well as of overall social role impairment.

These findings are particularly relevant as it had been claimed that irritability is to psychopathology what fever is to general medicine.^130^ This analogy may not apply for 2 reasons. First, in contrast to fever, irritability does not seem to be simply the generic expression of different types of problems, but to be related differentially to depression and anxiety in longitudinal and genetic studies. Second, irritability appears to be associated with future impairment even when adjusting for concurrent psychopathology, suggesting that, unlike most cases of pyrexia, it may have long-lasting consequences. However, it is clear that much work is required to better understand the position of irritability in nosology. Below we provide an outline of what we consider to be important next steps in the field.

### Measurement

Probably the main limitation in the study and treatment of irritability so far is the lack of high-quality measures. Most of the measurement of irritability has been done with instruments created ad hoc, that is, through extracting items from existing scales or interviews that were not intended primarily to measure irritability. This was an understandable approach, as it allowed researchers to exploit existing data. However, this approach risks overlooking important aspects of the phenotype in question, or measuring it with problematic reliability and validity, thereby reducing the signal-to-noise ratio. Future studies should make use of existing instruments specifically designed to capture irritability or develop new measures. To facilitate thinking about new approaches to measurement, we break down the section into different domains.

#### Questionnaires

Although it was not a measure specifically designed to capture irritability, the Multidimensional Assessment of Preschool Disruptive Behavior (MAP-DB) Questionnaire is a valid instrument to assess irritability features in preschool children. The MAP-DB Temper Loss subscale contains 20 items that assess several features of tantrums and anger regulation with good internal consistency (α = 0.97), providing a broad coverage of behaviors.^131^ The recently developed Affective Reactivity Index (ARI) is a concise way of capturing irritability with data on reliability and validity for both parent- and self-reported scales.^50^ It is short by design so as to allow busy clinicians to measure irritability in their patients and to be appropriate for use in large studies, in which participants are often asked several dozens to hundreds of questions. It allows dimensional measurement of irritability and is now being used in mechanistic studies, including brain imaging.^94^ However, researchers interested in systematically eliciting information about either specific symptoms related to irritability (e.g., angry rumination or examples of aggression), or in examining environmental influences (context of outbursts, interpersonal relationships) on irritability, will need to create new and possibly longer measures.

#### Change-Sensitive Measures

It is important to create interviews and questionnaires that are sensitive to change to monitor treatment outcomes, similar to what exists for OCD (Children’s Yale−Brown Obsessive Compulsive Scale) and depression (Children’s Depression Rating Scale). Parent-, self-, and clinician-reported versions of the ARI are currently being tested for this, and other measures should also be encouraged and tested in view of the need for treatment studies.

#### Observational Measures

Systematic observation can be used to capture behavioral aspects of irritability. This can include automated coding of facial expressions (FACS) using machine learning techniques. These measures have been shown to detect anxiety expressions in youth with ASD during stressful situations (unpublished data, August, 2015).

Finally, it is imperative to examine irritability using both multi-informant and multi-method studies. First, few studies have examined interrater reliability between parent and self reports,^38^, ^55^ and only one study has done so using an irritability measure.^50^ New instruments, including questionnaires and interviews, should incorporate different versions for parent and child. In addition, little is known about how teachers rate irritability. Second, the assessment of irritability can include other modalities of measurement, such as physiological (i.e., heart rate or skin conductance), biological (i.e., levels of cortisol),^120^ behavioral (i.e., eye-tracking), neurological (i.e., fMRI), and day-to-day monitoring using digital technology.

#### Experimental Paradigms

Reviews of paradigms designed to elicit changes in cortisol as a consequence of negative emotions such as fear or anger show that a substantial proportion of young people display no evidence of experiencing the stimulus as a stress challenge unless the task also evokes negative self-referent emotions such as shame and public exposure.^132^, ^133^ This probably also applies to anger-induction methods: those that include social exposure (i.e., the participant feels that he or she is being evaluated and compared with peers) and harassment (i.e., making negative comments about the participant’s task performance) appear more powerful in inducing self-reported anger and associated physiological responses.^134^ This complexity needs to be borne in mind when designing future anger induction tasks.

As mentioned in the introductory section of this article, the Research Domain Criteria’s frustrative nonreward may prove a very useful concept for the experimental study of irritability.^12^, ^98^ The basic concept is that of blocked goal attainment, or of the absence or delay of reinforcement,^135^ something that is readily manipulable in an experimental setup. This conceptual approach should appeal to clinicians who observe a child’s outburst when he or she is not given what he or she has been given to expect from parents or peers. However, frustrative nonreward in its own right may not be sufficient to explain why the proneness to anger persists beyond the specific events of blocked goal attainment. For this, further processes of instrumental learning will need to be investigated. More generally, there is good theoretical background^136^, ^137^ and emerging empirical evidence^98^ for investigating anger and irritability within a reward framework. This has been a fruitful strategy in depression research,^138^ and informs the broader field of behavioural neuroeconomics and decision making.^139^

#### Treatment

To date, there is only 1 RCT of medication for children with SMD. No pharmacological studies have been done to examine the treatment outcomes of children with DMDD, which is understandable, given that it was only recently introduced. So far, evidence shows that irritability and anger respond to different treatments within the context of different disorders; for example, irritability responds to stimulants intended to treat hyperactivity and inattention in children with ADHD, and irritability also seems to respond to exposure treatment for anxiety in children with OCD. However, irritability was not the primary outcome in many studies, and some results are extracted from post hoc analyses of available data. The observation that many different treatments primarily used for other disorders appear to benefit moderately those with irritability can have 2 explanations. First, treatments such as stimulants may target a set of mechanisms that are generic and part of various different disorders. Second, irritability may improve because it is secondary to the disorder being treated; for example, once ADHD symptoms improve, irritability may also reduce. As mentioned above, validating instruments for the sensitive measurement of change will be a crucial step toward designing better treatment studies with irritability as primary outcome. In addition, given the nonspecific response of irritability to different treatments in the context of distinct disorders, future studies must examine whether these treatments influence irritability before, at the same time, or after symptoms of the primary disorder.

It is an obvious aim to test parenting interventions—which are known to be useful in ODD^140^—in irritability. More generally, the role of parent–child relationships in the development of irritability and the possible protective effects of parental appraisals^141^ should be tested.

#### Validity

We systematically reviewed data on the validity of the irritability construct based on factor analyses, longitudinal stability and consistency, longitudinal correlates, and etiological underpinnings. Although evidence to date allows us to draw some conclusions, these are limited for several reasons.

First, factor and latent class analyses have been conducted within a narrow set of items usually derived post hoc (mainly by using ODD symptom measures). These analyses should be replicated using a larger set of variables including more irritability variables, ideally as part of well-validated instruments, and items assessing symptoms from other psychiatric disorders (e.g. ADHD or depression symptoms). In addition, multivariate studies show that the correlation between the ODD dimensions of irritability and headstrong ranges from moderate^38^, ^40^, ^142^, ^143^ to high.^36^ Understanding the causes of such variation will be important to disentangle the unique and generic pathophysiological factors contributing to irritability.

Second, our review and meta-analysis of longitudinal correlates of irritability pointed toward depression and anxiety as its main future correlates. However, there was heterogeneity among studies. Although most studies used dimensional measures of irritability, some studies used categorical definitions.^30^, ^31^, ^57^ Moreover, instruments used to measure irritability differed between studies. In addition, only some studies adjusted analyses for the presence of headstrong/hurtful symptoms, which is important given the high correlations between these dimensions. Studies also differed in adjustments for baseline psychopathology, demographic factors such as sex and age, or socioeconomic status. Despite these differences, however, analyses of subgroups and meta-regressions did not yield significant results. We were able to test for publication bias only in anxiety and depression due to the low number of studies in the other domains. We did not find evidence for publication bias in anxiety. In depression, there was weak evidence for publication bias, which is probably best explained by the heterogeneity of the studies included. Indeed, after two studies contributing to heterogeneity were excluded, there was no longer evidence of bias. Finally, most studies examining longitudinal correlates included depression as an outcome, but very few included BD, conduct problems, and substance abuse. For future research, more studies predicting several outcomes, using different cohorts, and deriving from independent research groups are needed.

Regarding etiological underpinnings, there is evidence on the heritability of irritability, yet there are very few studies addressing its genetic association with other disorders, especially other than depression.^37^, ^69^

Behavioral and neuroimaging research on chronic irritability is a newly emerging field. Mapping between behavioral and imaging findings is still outstanding, and so far only 1 published neuroimaging study has used continuous measures of irritability.^94^ In some studies, it has been difficult to separate the effects of ODD or ADHD on irritability findings because of high comorbidity with SMD. Moreover, behavioral and neuroimaging studies have focused on differentiating SMD from BD. It is imperative to include other comparison groups such as depression, anxiety, ODD, ADHD, or ASD to examine their shared and distinct underpinnings. The developmental pathway from irritability to depression and their genetic overlap is one of the main findings in this review. This should prompt future neurobiological studies that examine the possible substrates for this overlap by testing experimental paradigms that incorporate transitions between cardinal emotions such as anger and sadness, for example.

In this review, we have not separately analyzed DMDD and SMD, as doing so would lead to very small groups to analyze with validity. It is important to note that the vast majority of youth with SMD meet criteria for DMDD. For example, in a report by Deveney *et al.*,^58^ of the 200 youth who met criteria for SMD, 97% met criteria for DMDD; the 6 youth who did not meet criteria for DMDD had an age of onset of 10 or 11 years old. Similarly, in a report by Stoddard *et al.*,^95^ all but 1 youth with SMD met criteria for DMDD. It therefore seems reasonable to pool these 2 diagnoses in our analyses. Also, following the current conceptualization of psychiatric disorders as extremes of dimensional traits,^16^ we regard DMDD and SMD as lying above a certain threshold on an irritability continuum. This is an assumption supported by prior data on irritability,^131^ although not DMDD or SMD as such. Future research should test the validity of that assumption also in terms of underlying pathophysiological mechanisms.

**Clinical Guidance**

• Our review showed that irritability is impairing for young people and that it is associated with a substantially increased likelihood of future psychopathology, particularly depression and anxiety.
• In understanding how irritability arises and when it becomes pathological, a lot has been accomplished within a short period of time. However, a number of areas—most notably measurement and experimental design—still require research attention.
• Clinicians will want to measure irritability in their patients as outlined in this review and apply the—admittedly still scarce—evidence in treating it.
