# Supplementary material for: The Status of Irritability in Psychiatry: A Conceptual and Quantitative Review
Source: J Am Acad Child Adolesc Psychiatry. 2016 Jul;55(7):556–70. doi: 10.1016/j.jaac.2016.04.014 (PMC4927461; doi:10.1016/j.jaac.2016.04.014)
Supplement: Supplemental Material [file mmc1.docx]

**SUPPLEMENT 1**

In this section we provide the methodology of the meta-analysis as well as additional analyses and results.

**METHOD**

**Data Sources and Search Strategies:**

We searched PubMed and Web of Science through December 2014 (updated February 2015) using the terms “irritability,” “irritable,” “disruptive mood dysregulation,” “severe mood dysregulation,” “oppositional” or “ODD,” together with “dimension,” “subdimension,” “class,” “factor” or “subfactor” AND “predict,” “longitudinal,” and “prospective.” No limits were applied for language or date of publication. All articles obtained from the search were then manually assessed for inclusion or exclusion based on the presence of search terms by two independent investigators. In addition, the reference list of each relevant article was reviewed, as well as papers citing these articles in Google Scholar.

**Inclusion and Exclusion Criteria:**

We included longitudinal studies where chronic non-episodic irritability was the predictor of future psychopathology.

For the purposes of this meta-analysis, chronic non-episodic irritability was defined as a dimensional measure of irritability based on criteria defining oppositional defiant disorder (ODD) symptomatology, or alternatively, as a categorical measure like disruptive mood dysregulation disorder (DMDD) or severe mood dysregulation disorder (SMD). On the other hand, studies where episodic irritability was the predictor (e.g., in the context of depression disorder, bipolar disorder, or premenstrual syndrome) were excluded.

Future psychopathology was defined as any type of psychiatric disorder identified through well validated and (semi-)structured interviews, or otherwise psychopathological scores in well-validated measures.

Prospective, longitudinal studies were included if they were published in peer-reviewed journals. No limits were applied to the age of participants at baseline or the years of follow-up, nor to the type of sample (i.e., community-based or clinical).

**Study Selection**

Our search strategy results are shown in **Figure 1**. The search returned 163 articles after duplicates were removed, for which the title were reviewed for relevance. Following this review, 67 articles were excluded. After a second review, 72 further articles were excluded based on the following exclusion criteria: 6 articles were cross-sectional; 1 article was retrospective; 10 articles were focused on episodic irritability; in 13 articles, irritability was the outcome; in 23 articles, outcome was other than psychopathology; in 10 articles, the outcome was psychopathology but irritability was not the predictor; 9 articles were actually reviews. The reference sections and citations in Google Scholar of the remaining 24 studies were assessed for other potentially relevant articles, which yielded 3 additional studies to be included. Therefore, 27 studies were included in the full-text review. After this last review, conducted by two investigators, 3 studies were excluded for using an idiosyncratic definition of irritability. The search and review of studies resulted in 12 studies appropriate for inclusion in the meta-analysis for the prediction of psychiatric disorders,[^1-12^](#_ENREF_1) and 12 studies predicting continuous outcomes [^13-22^](#_ENREF_13) or appropriate for descriptive analysis.[^23^](#_ENREF_23)^,^ [^24^](#_ENREF_24) When possible, studies predicting continuous outcomes were subjected to a separate meta-analysis and pooled standardized beta coefficients and 95% CIs were also calculated. Otherwise, only a description of the findings is provided.

**Data Extraction**

For the 12 studies predicting psychiatric disorders, outcomes representing the impact of irritability on future psychopathology were presented in odds ratio (OR) and 95% CI. Study characteristics were extracted to account for heterogeneity among studies. These data included (a) type of measured irritability (dimensional or categorical) and how it is defined (i.e. ODD dimension, DMDD, or SMD) (b) participant characteristics controlled (i.e., age, sex, family socioeconomic status [SES]); (c) whether baseline levels of psychopathology were controlled, including symptoms comprising the headstrong dimension; (d) ages at baseline and follow-up, and years between baseline and follow-up assessments; and (e) type of sample (clinical or community), geographic origin of the sample (US or other countries), cohort, and proportion of males in the sample.

**Data Analysis**

Given that the studies predicting psychiatric disorders included in the meta-analysis varied in methodology and design, a random-effects model was calculated using Stata 11. We conducted the meta-analysis for findings where outcome data from two or more studies of different cohorts could be combined; this included the prediction of depression, anxiety disorder, bipolar disorder, attention-deficit/hyperactivity disorder (ADHD), oppositional defiant disorder (ODD), conduct disorder (CD) (antisocial personality disorder in adulthood), and substance disorder. We calculated pooled ORs for each of these outcomes.

When two or more studies from the same cohort predicted the same outcome, each of these studies was individually excluded from the analysis, and the pooled OR and 95% CI were recalculated. If an individual study from a specific cohort contributed heavily to the pooled OR, a change in the magnitude or significance of the pooled OR would be observed.


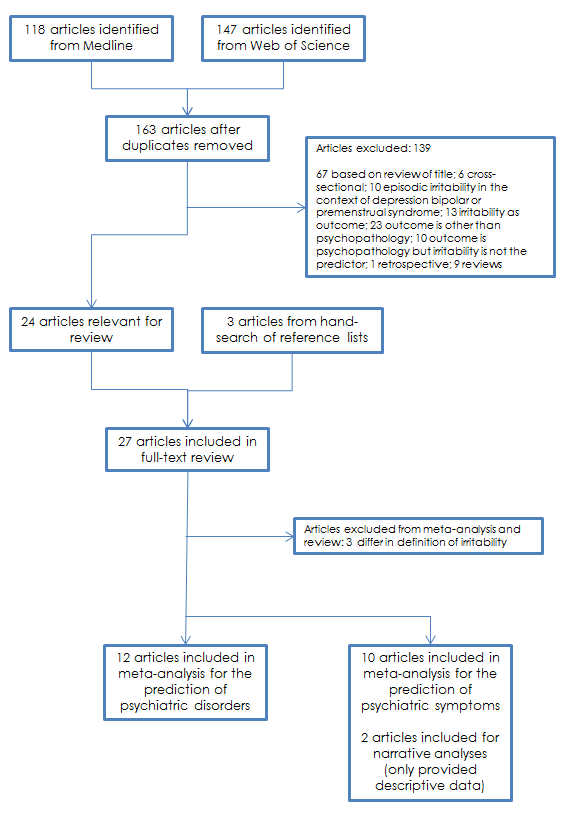


**FIGURE S1.** Review and selection of articles for meta-analysis.

We tested for between-study heterogeneity using the *I*^2^ statistic, which is the percentage of variation attributable to heterogeneity. The values of *I*^2^ lie between 0% and 100%, with larger values showing increasing heterogeneity. Higgins et al [^25^](#_ENREF_25) suggest that *I*^2^ values between 25%-50% are low, moderate for 50%-75%, and high for ≥75%.

Subgroup analyses with categorical covariates and meta-regression with continuous covariates were employed to explore sources of heterogeneity.

Given the possibility of publication bias (i.e., significant findings are more likely to be published), we examined whether there was asymmetry in funnel plots (a scatterplot of the estimates from individual studies against a measure of a study size) and calculated the Egger’s coefficient bias. Egger’s test is a regression-based test for formally detecting skewness in the funnel plots and, therefore, publication bias in the data.[^26^](#_ENREF_26) This test evaluates whether the intercept deviates significantly from zero in a regression of standardized effect estimates against their precision. It is recommended that test for publication bias in meta-analysis should only be used when there are 10 or more studies, especially if there is substantial heterogeneity between studies.[^27^](#_ENREF_27) This is because tests power is usually too low to distinguish chance from real asymmetry in funnel plots.

**Additional Analyses and Results**

In this section we present the results of excluding individual studies of the same cohort and recalculate the pooled OR and 95%CI for each outcome. We only provide the range from the lower OR to the higher OR as well as from the lower to the higher heterogeneity (*I*^2^).

The study of Stringaris and Goodman[^12^](#_ENREF_12) used internalizing disorder as outcome, which combined both depressive and anxiety disorders. Therefore, this study was included in both analyses, for the prediction of both depressive and anxiety disorders. Pooled effect sizes were recalculated for each of these analyses after removing this study.

**Irritability as a Predictor of Psychiatric Disorders**

*Depressive Disorder*: Recalculated pooled OR after removing studies from the same cohort ranged from OR= 1.57, 95% CI 1.31-1.89 to OR=2, 95% CI 1.58-2.36 (all *p*<.001), with between-study heterogeneity ranging from low (*I*^2^=0%) to moderate (*I*^2^=44.8%). Removing Stringaris and Goodman,[^12^](#_ENREF_12) which predicted internalizing disorders, did not alter the results significantly (OR=1.83, 95% CI=1.40-2.40, *p*<.001).

*Anxiety Disorders:* Meta-analyses performed after removing studies from the same cohort revealed that the whole heterogeneity was explained by the prediction of Copeland et al.[^4^](#_ENREF_4) When this study was included, pooled OR ranged from 1.81, 95% CI 1.18-2.77, *p*=.007 to 1.90, 95% CI 1.28-2.83, *p*=.002, with an *I*^2^ of 68.3% and 68.5%, respectively. However, when this study was not included, OR ranged from 1.43, 95% CI 1.17-1.76 to 1.55, 95% CI 1.32-1.82 (all *p*≤.001) with an *I*^2^ of 0% in all cases. Finally, removing Stringaris and Goodman,[^12^](#_ENREF_12) which predicted a combination of depressive and anxiety disorders, did not alter the results significantly (OR=1.73, 95% CI=1.25-2.39, *p*=.001).

*Bipolar Disorder*: Removing studies from the same cohort did not change the results (all *p*>.9; *I*^2^=0%).

*Conduct Disorder*: When removing studies from the same cohort, results ranged from OR=0.87, 95% CI 0.62-1.21, *p*=.400, *I*^2^=0% to OR=0.97, 95% CI 0.70-1.34, *p*=.887, *I*^2^=37.5%.

*ODD***:** When removing the study of Brotman et al.[^3^](#_ENREF_3) from the Great Smoky Mountains cohort, the overall effect size was OR=2.59, 95% CI=1.37-4.90, *p*=.003, *I*^2^=86.5%, whereas when removing the study of Rowe et al.[^10^](#_ENREF_10) the pooled effect size was OR=3.53, 95% CI=1.33-9.37, *p*=.011, *I*^2^=85.2%.

The high between-study heterogeneity in the prediction of ODD was totally explained by two studies. The first of these studies was Ezpeleta et al.,[^6^](#_ENREF_6) who compared children with a trajectory of high persistent irritability to children without irritability. Given that the sample size of the first group was small (n=23) and the prevalence of ODD at follow-up was much higher in comparison to children without irritability (58.4% vs 3.36%), this resulted in an outlier value in the prediction of ODD (OR=81.88, 95% CI=14.68-456.69, *p*<.05). In addition, this study did not control for baseline rates of ODD. The second study contributing to heterogeneity was the single clinical study,[^7^](#_ENREF_7) which consisted of a clinical trial of patients with either CD or ODD. Removing these two studies from the analysis resulted in a heterogeneity of *I*^2^=0% with a pooled OR of 1.57, 95% CI=1.28-1.92, *p*<.001.

*Substance Abuse/Dependence*: When removing studies from the same cohort, results ranged from OR=0.21, 95% CI 0.59-2.47, *p*=.608, *I*^2^=71.6% to OR=1.71, 95% CI 0.95-3.09, *p*=.076, *I*^2^=0%.

To explore whether the covariates could account for some of the variability among the effect sizes for the prediction of depression, anxiety, and ODD, we conducted subgroup analyses where separate OR and p values were calculated for each level of the categorical covariates. Meta-regressions were used to explore continuous covariates. Given small number of studies predicting each outcome, these results are intended to be hypothesis-generating as opposed to identifying conclusively reasons for heterogeneity among studies.

For the prediction of depression, no significant differences in OR were found for any covariate between sub-groups. However, the pooled ORs of those studies that adjusted for headstrong symptoms (n=2) (1.42, 95% CI 097-2.07, p=.068)[^10^](#_ENREF_10)^,^ [^12^](#_ENREF_12) as well as those studies that did not adjust for sex (n=2) (2.90, 95% CI 0.53-15.96, p=.222)[^2^](#_ENREF_2)^,^ [^4^](#_ENREF_4) were not significant.

For the prediction of anxiety, no significant differences in OR were found for any covariate between sub-groups. However, the pooled ORs of those studies that employed categorical definitions of irritability (i.e., DMDD or SMD) (n=5) (2.08, 95% CI 0.83-5.23, p=.120), as well those that did not adjust for baseline disorder (n=4) (2.39, 95% CI 0.85-6.69, p=.098), age (n=5) (2.10, 95% CI 0.82-5.36, p=.122), or sex (n=3) (2.95, 95% CI 0.72-11.98, p=.131) were not significant.

Finally, for the prediction of ODD, as in the other cases, no differences in OR were found for any covariate between sub-groups. However, the pooled OR of those studies that employed categorical definitions of irritability (n=3) (6.64, 95% CI 0.29-152.47, p=.236) was not significant.

Analyses of continuous covariates for each prediction were not significant.

**Test for Publication Bias**

In our meta-analysis, only the pooled ORs of two outcomes were based in 10 studies: depression and anxiety. Therefore, we tested whether it might be publication bias of studies predicting depression and anxiety from chronic irritability.

**Figure S2a** shows the funnel plot of the 10 studies predicting depression. Two studies in the lower right of the funnel, Brotman et al[^3^](#_ENREF_3) and Copeland et al,[^4^](#_ENREF_4) contributed to asymmetry. The Egger’s bias coefficient also suggested the presence of asymmetry and publication bias (bias=2.60, p=.004). However, since most of the individual effect estimates were above zero, the effect of publication bias, if any, would be to inflate the estimate rather than to lead to an incorrect conclusion about the existence of an effect. Moreover, when removing these 2 studies from the analyses, not only the pooled OR remained significant and heterogeneity decreased (OR=1.61, 95% CI 1.35-1.93, *p*=.076, *I*^2^=34.6%.), but the Egger’s bias coefficient was no longer significant (bias=2.21, p=.067). As can be seen in **Figure S2b**, the funnel plot after excluding these 2 studies showed more symmetry. These 2 studies provided larger OR and larger variance that the remaining studies. This could be explained because these were the only studies that captured irritability categorically based on SMD or DMDD criteria in a community sample—the same sample. Therefore, prevalence of positive cases was very low (i.e., 3.3% in Brotman et al[^3^](#_ENREF_3) and 4.1% in Copeland et al[^4^](#_ENREF_4)) compared with the negative cases (>90%), then leading to large OR and variances.

**
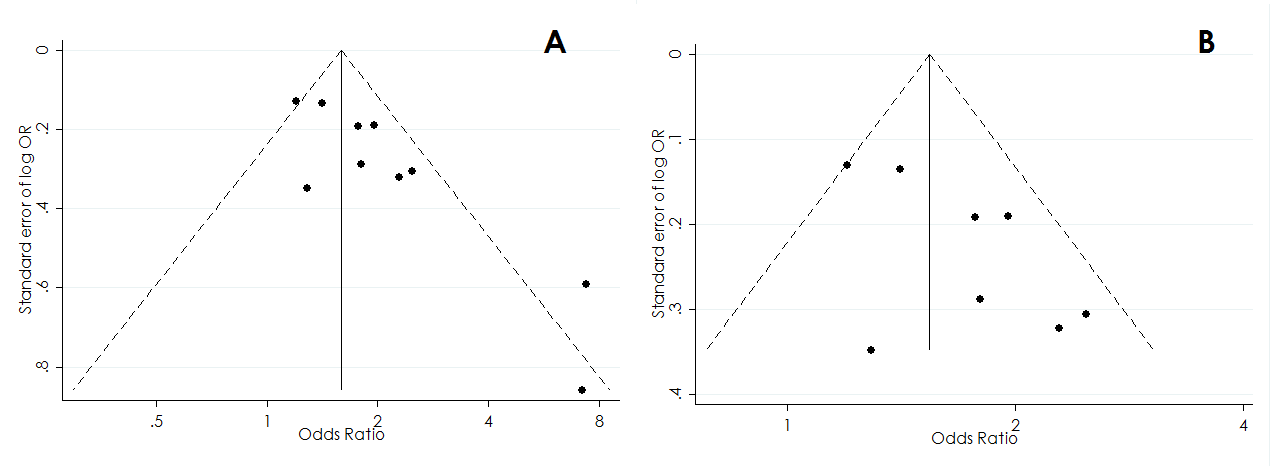
**

**FIGURE S2.** Funnel plot with pseudo 95% confidence limits, using data from 10 studies predicting depression (A) and 8 of these studies after excluding outlier estimates (B).

In the prediction of anxiety, only the study from Copeland et al[^4^](#_ENREF_4) was in the mid-right region outside the funnel (**Figure S3**). Overall, the funnel plot shows symmetry across studies, and the Egger’s bias coefficient also suggested the absence of asymmetry and publication bias (bias=0.62, *p*=.536).

**
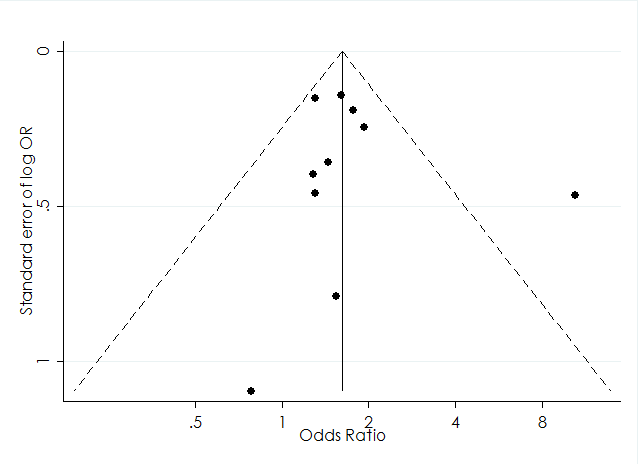
**

**FIGURE S3.** Funnel plot with pseudo 95% confidence limits, using data from 10 studies predicting anxiety disorder.

It is important to note that the results of these tests should be taken carefully due to the number of individual studies included in this meta-analysis. There were only 10 studies, 8 when excluding outliers, and the pooled OR had moderate heterogeneity. Therefore, more studies are needed to conclude whether there is publication bias or not. In addition, publication bias is only one of many sources of asymmetry found in funnel plots.[^26^](#_ENREF_26)^,^ [^27^](#_ENREF_27) Test of publication bias in the prediction of the remaining outcomes, although these included less than 10 studies, yielded non-significant results.

**Prediction of Continuous Outcomes**

Among the 10 studies predicting continuous outcomes, 6 had depression symptoms as outcome, [^14^](#_ENREF_14)^,^ [^15^](#_ENREF_15)^,^ [^17^](#_ENREF_17)^,^ [^20-22^](#_ENREF_20) and 4 studies predicted internalizing symptoms, which included depression and anxiety symptoms.[^13^](#_ENREF_13)^,^ [^16^](#_ENREF_16)^,^ [^18^](#_ENREF_18)^,^ [^19^](#_ENREF_19) All these studies but one significantly predicted depression or internalizing symptoms. The prediction in the Leadbeater and Homel study was only significant from ages 18-19 to ages 20-21.[^18^](#_ENREF_18) Pooled estimates for the prediction of depression symptoms based on 6 studies yielded significant results (pooled standardized beta coefficient= 0.14, 95%CI= 0.09, 0.20, p<.001 ) as did the pooled estimates of 3 studies for the prediction of internalizing symptoms (pooled standardized beta coefficient= 0.21, 95%CI= 0.06, 0.19, p<.001). There was substantial heterogeneity across these studies (*I*^2^=82.7% and *I*^2^=83.6%, respectively), probably because of differences in analytic approaches, methodology, and design. The authors of one study[^13^](#_ENREF_13) were contacted for further information in order to pool their data with that from the other studies but did not reply; however, the prediction of internalizing disorders in this study[^13^](#_ENREF_13) was significant (ES=0.21, p<.05).

Two further studies predicted anxiety symptoms separately,[^14^](#_ENREF_14)^,^ [^17^](#_ENREF_17) but only one found significant results (ES=0.12).[^14^](#_ENREF_14) In addition, a further study found that rates of depression and anxiety increased at 2 and 4 years follow-up in a sample of youth with SMD,[^23^](#_ENREF_23) whereas rates of ODD and ADHD were high but unchanged.

There are few studies with bipolar disorder or mania as outcome. Although the outcome was not measured continuously, here we describe studies that provide rates of future bipolar disorder in youth with chronic irritability. In a referred sample, 84 youths with SMD and 93 youths with *DSM-IV* bipolar disorder were followed over a median of 28.4 months. At follow-up only one patient (1.2%) with SMD exhibited one or more manic, hypomanic, or mixed episodes, compared to 58 patients (62.4%) with bipolar disorder.[^24^](#_ENREF_24) Similarly, no children with SMD at age 10 had bipolar disorder at age 18 (Brotman et al.[^3^](#_ENREF_3)), and only 1 participant developed bipolar disorder at 4-year follow-up in a sample of 200 children with SMD.[^23^](#_ENREF_23)

Five studies have tested the longitudinal association between chronic irritability and conduct problems,[^15^](#_ENREF_15)^,^ [^18^](#_ENREF_18)^,^ [^21^](#_ENREF_21) externalizing symptoms,[^19^](#_ENREF_19) or callous unemotional traits.[^13^](#_ENREF_13)^,^[^15^](#_ENREF_15)^,^[^16^](#_ENREF_16)^,^[^18^](#_ENREF_18)^,^[^22^](#_ENREF_22) Of those, only two found a significant association between baseline irritability and callous unemotional traits[^13^](#_ENREF_13) at follow up.[^13^](#_ENREF_13)^,^ [^15^](#_ENREF_15) The pooled estimate for conduct problems was not significant (pooled standardized beta coefficient= -0.01, 95%CI= -0.14, 0.13; p= .942, *I*^2^=67%).

Although criminal behaviors are not strictly speaking psychiatric disorders, four studies examined how this was predicted by irritability.[^1^](#_ENREF_1)^,^ [^4^](#_ENREF_4)^,^ [^20^](#_ENREF_20)^,^ [^28^](#_ENREF_28) Only the study with categorically defined irritability (i.e., DMDD) found a significant association.[^4^](#_ENREF_4) In contrast, studies examining ODD dimensions found that the headstrong/hurtful dimension of ODD, but not irritability, predicted criminal offences.[^1^](#_ENREF_1)^,^ [^4^](#_ENREF_4)^,^ [^20^](#_ENREF_20)^,^ [^28^](#_ENREF_28)

| **TABLE S1.** Characteristics of Studies Predicting Psychiatric Disorders Included in the Meta-Analysis | | | | | | | | |
| --- | --- | --- | --- | --- | --- | --- | --- | --- |
| **Study (Cohort)** | **N** | **Sex**  **(males)** | **Baseline age^a^** | **Follow-up age^a^** | **Baseline assessment of irritability** | **Type of irritability** | **Outcomes** | **Follow-up assessment of psychiatric disorder** |
| Althoff et al. 2014[^1^](#_ENREF_1) (Zuid-Holland Longitudinal Study) | 49 | 49% | 3-17 years | 18-32 years | Parent report on Child Behavior Checklist | ODD symptoms | Any mood disorder | Self-report on the Composite International Diagnostic Interview |
| Axelson et al. 2012 [^2^](#_ENREF_2) (Longitudinal Assessment of Manic Symptoms) | 433 | 67% | 6-12 years | 8-14 years | Parent report on the Schedule for Affective Disorders and Schizophrenia for School-Age Children-Present and Lifetime Version | DMDD | Bipolar spectrum  Any depressive disorder  Anxiety disorder  Conduct disorder | Parent report on the Schedule for Affective Disorders and Schizophrenia for School-Age Children-Present and Lifetime Version |
| Brotman et al. 2006[^3^](#_ENREF_3) (Great Smoky Mountains) | 1,420 | 56% | 9-13 years | 16-22 years | Parent report on the Child and Adolescent Psychiatric Assessment | SMD | Any depressive  Any anxiety  ADHD  CD  ODD  Substance disorder | Self-report on the Young Adult Psychiatric Assessment |
| Copeland et al. 2014[^4^](#_ENREF_4) (Great Smoky Mountains) | 1,273 | 56% | 9-13 years | 24-26 years | Parent report on the Child and Adolescent Psychiatric Assessment | DMDD | Depressive  Anxiety  ASPD  Alcohol | Self-report on the Young Adult Psychiatric Assessment |
| Dougherty et al. 2013[^5^](#_ENREF_5) (Community close to Stony Brook University, NY) | 462 | 54% | 3.6 years | 6.1 years | Parent report on the Preschool Age Psychiatric Assessment | ODD symptoms | Depressive  Anxiety  ADHD  ODD | Parent report on the Preschool Age Psychiatric Assessment |
| Ezpeleta et al. 2015[^6^](#_ENREF_6) (Community in Barcelona) | 218 | 50% | 3.8 years | 6 years | Parent report on the Diagnostic Interview of Children and Adolescents for Parents of Preschool Children | ODD symptoms  dimension | ADHD  Anxiety  ODD | Parent report on the Diagnostic Interview of Children and Adolescents for Parents of Preschool Children |
| Kolko et al. 2010[^7^](#_ENREF_7) (Patients from University of Pittsburgh Medical Center) | 177 | 84% | 6-11 years | 9-14 years | Parent and child report on the Schedule for Affective Disorders and Schizophrenia for School-Age Children | ODD symptoms  dimension | ADHD  CD  ODD | Parent report on the Schedule for Affective Disorders and Schizophrenia for School-Age Children |
| Leibenluft et al. 2006[^8^](#_ENREF_8) (Community in New York) | 776 | 50% | 9-19 years | 17-28 years | Parent and child report on the Diagnostic Interview Schedule for Children | ODD symptoms | MDD  GAD  ADHD  CD  ODD  Mania | Self-report on the Diagnostic Interview Schedule for Children |
| Pickles et al. 2010[^9^](#_ENREF_9) (Isle of Wight) | 2,226 | 50% | 14-15 years | 44-45 years | Parent and child report on a structured interview | SMD-like | MDD  GAD  Substance disorder | Self-report on Schedule for Affective Disorders and Schizophrenia |
| Rowe et al. 2010[^10^](#_ENREF_10) (Great Smoky Mountains) | 1,420 | 56% | 9-13 years | 16 years | Parent report on the Child and Adolescent Psychiatric Assessment | ODD symptoms  dimension | Depression  anxiety  CD  ODD  Substance disorder | Parent report on the Child and Adolescent Psychiatric Assessment |
| Stringaris et al. 2009[^11^](#_ENREF_11) (Community in New York) | 776 | 46% | 13.8 years | 33.2 years | Parent and child report on the Diagnostic Interview Schedule for Children | ODD symptoms | MDD  GAD  Bipolar disorder | Self-report on the Structured Clinical Interview for DSM-IV Axis I Disorders |
| Stringaris et al. 2009[^12^](#_ENREF_12) (British Child and Adolescent Mental Health Survey) | 1,833 | 59% | 9.8 years | 12.8 years | Parent report on the Development and Well-Being Assessment | ODD symptoms  dimension | Internalizing disorders (Depressive + Anxiety)  ADHD  CD | Parent report on the Development and Well-Being Assessment |
| Note: ADHD = attention-deficit/hyperactivity disorder; ASPD = antisocial personality disorder; CD = conduct disorder; DMDD = disruptive mood dysregulation disorder; GAD = generalized anxiety disorder; MDD = major depressive disorder; ODD = oppositional defiant disorder; SMD = severe mood dysregulation.  ^a^ Age: Range, or mean if range not indicated | | | | | | | | |

| **TABLE S2.** Characteristics of Studies Predicting Psychiatric Symptoms | | | | | | | | |
| --- | --- | --- | --- | --- | --- | --- | --- | --- |
| **Study (Cohort)** | **N** | **Sex**  **(males)** | **Baseline age^a^** | **Follow-up age^a^** | **Baseline assessment of irritability** | **Type of irritability** | **Outcomes** | **Follow-up assessment of psychiatric symptoms** |
| Burke et al. 2010[^15^](#_ENREF_15)  (Pittsburgh Girls Study) | 2451 | 0% | 5-8 years | 11.5 years | Self- report on Child Symptom Inventory – IV | ODD symptoms | Depressive symptoms  Conduct symptoms | Self- report on Child Symptom Inventory – IV |
| Stringaris et al. 2012[^20^](#_ENREF_20)  (G1219) | 1597 | 44% | 12-12 years | 14-23 years | Youth Self-Report (for ages 11–18) and the Adult Self-Report (for ages 18–59) of the ASEBA family of instrument. | ODD symptoms | Depressive symptoms  Delinquent behavior | Depressive symptoms – Self-report on Short Mood and Feelings Questionnaire  Delinquent behavior- Self-report on ASEBA |
| Burke et al. 2012[^14^](#_ENREF_14)  (Developmental Trends Study) | 165 | 100% | 7-12 years | 17 years | Parent report on the Diagnostic Interview Schedule for Children | ODD symptoms | Depressive symptoms  Anxiety symptoms | Parent report on the Diagnostic Interview Schedule for Children |
| Whelan et al. 2013[^22^](#_ENREF_22)  (Avon Longitudinal Study of Parents and Children) | 6328 | 51% | 13 years | 16 years | Parent report on the Development and Well Being Assessment | ODD symptoms | Depressive symptoms  Conduct symptoms | Depressive symptoms – Self-report on Short Mood and Feelings Questionnaire  Conduct symptoms – Parent report on the Strengths and Difficulties Questionnaire |
| Leadbeater et al 2015[^18^](#_ENREF_18)  (Victoria Healthy Youth Survey) | 464 | 48% | 22-23 years | 24-25 years | Self-report on Brief Child and Family Phone Interview | ODD symptoms | Internalizing symptoms  Conduct symptoms | Self-report on Brief Child and Family Phone Interview |
| Whelan et al 2015[^21^](#_ENREF_21)  (Avon Longitudinal Study of Parents and Children) | 3963 | 41% | 8 years | 13 years | Parent report on the Development and Well Being Assessment | ODD symptoms | Depressive symptoms  Conduct symptoms | Depressive symptoms – Self-report on Short Mood and Feelings Questionnaire  Conduct symptoms – Parent report on the Strengths and Difficulties Questionnaire |
| Lavigne et al. 2014 | 796 | 49% | 4.4 years | 6.5 years | Parent report on Diagnostic Interview Schedule for Children (DISC)-Parent Scale Young Child | ODD symptoms | Depressive symptoms  Anxiety symptoms | Parent report on DISC and Child Symptom Inventory |
| Barker et al. 2012[^13^](#_ENREF_13)  (Avon Longitudinal Study of Parents and Children) | 5923 | 51% | 8 years | 13 years | Parent report on the Development and Well Being Assessment | ODD symptoms | Internalizing symptoms  Callous-unemotional traits | Parent report on the Development and Well Being Assessment |
| Herzhoff and Tacket 2015[^16^](#_ENREF_16) | 439 | 49% | 9-10 years | 11-12 years | Parent report on Computerized Diagnostic Interview Schedule for Children | ODD symptoms | Internalizing symptoms  Externalizing symptoms | Parent report on Child Behavior Checklist |
| Savage et al. 2015[^19^](#_ENREF_19)  (Swedish Twin Study of Child and Adolescent Development) | 576 | - | 16-17 years | 19-20 years | Parent report on Child Behavior Checklist | ODD symptoms | Anxious/depressed symptoms | Self-report on Adult Behavior Checklist |
| Stringaris et al. 2010[^24^](#_ENREF_24) | 84 | 67% | 11.6 years | 13.6 years | Parent and child report on The Kiddie Schedule for Affective Disorders—Present  and Lifetime Version | SMD | Manic episodes  Depressive episodes | Parent and child report on The Kiddie Schedule for Affective Disorders—Present  and Lifetime Version |
| Deveney et al. 2015[^23^](#_ENREF_23) | 200 |  | 7-17.6 years | 11-21 years | Parent and child report on The Kiddie Schedule for Affective Disorders—Present  and Lifetime Version | SMD | Depression disorder  Anxiety disorder  ADHD  ODD | Parent and child report on The Kiddie Schedule for Affective Disorders—Present  and Lifetime Version |
| Note: ADHD: Attention deficit hyperactivity disorder; ASEBA = Achenbach System of Empirically Based Assessment; ODD = oppositional defiant disorder; SMD = severe mood dysregulation. ^a^ Age: Range, or mean if range not indicated | | | | | | | | |

**REFERENCES**

**1.** Althoff RR, Kuny-Slock AV, Verhulst FC, Hudziak JJ, van der Ende J. Classes of oppositional-defiant behavior: concurrent and predictive validity. *J Child Psychol Psychiatry.* 2014;55:1162-1171.

**2.** Axelson D, Findling RL, Fristad MA, et al. Examining the proposed disruptive mood dysregulation disorder diagnosis in children in the Longitudinal Assessment of Manic Symptoms study. *J Clin Psychiatry.* 2012;73:1342-1350.

**3.** Brotman MA, Schmajuk M, Rich BA, et al. Prevalence, clinical correlates, and longitudinal course of severe mood dysregulation in children. *Biol Psychiatry.* 2006;60:991-997.

**4.** Copeland WE, Shanahan L, Egger H, Angold A, Costello EJ. Adult diagnostic and functional outcomes of DSM-5 disruptive mood dysregulation disorder. *Am J Psychiatry.* 2014;171:668-674.

**5.** Dougherty LR, Smith VC, Bufferd SJ, et al. Preschool irritability: longitudinal associations with psychiatric disorders at age 6 and parental psychopathology. *J Am Acad Child Adolesc Psychiatry.* 2013;52:1304-1313.

**6.** Ezpeleta L, Granero R, de la Osa N, Trepat E, Domenech JM. Trajectories of Oppositional Defiant Disorder Irritability Symptoms in Preschool Children. *J Abnorm Child Psychol.* 2015;44:115-128.

**7.** Kolko DJ, Pardini DA. ODD dimensions, ADHD, and callous-unemotional traits as predictors of treatment response in children with disruptive behavior disorders. *J Abnorm Psychol.* 2010;119:713-725.

**8.** Leibenluft E, Cohen P, Gorrindo T, Brook JS, Pine DS. Chronic versus episodic irritability in youth: a community-based, longitudinal study of clinical and diagnostic associations. *J Child Adolesc Psychopharmacol.* 2006;16:456-466.

**9.** Pickles A, Aglan A, Collishaw S, Messer J, Rutter M, Maughan B. Predictors of suicidality across the life span: the Isle of Wight study. *Psychol Med.* 2010;40:1453-1466.

**10.** Rowe R, Costello EJ, Angold A, Copeland WE, Maughan B. Developmental pathways in oppositional defiant disorder and conduct disorder. *J Abnorm Psychol.* 2010;119:726-738.

**11.** Stringaris A, Cohen P, Pine DS, Leibenluft E. Adult outcomes of youth irritability: a 20-year prospective community-based study. *Am J Psychiatry.* 2009;166:1048-1054.

**12.** Stringaris A, Goodman R. Longitudinal outcome of youth oppositionality: irritable, headstrong, and hurtful behaviors have distinctive predictions. *J Am Acad Child Adolesc Psychiatry.* 2009;48:404-412.

**13.** Barker ED, Salekin RT. Irritable oppositional defiance and callous unemotional traits: is the association partially explained by peer victimization? *J Child Psychol Psychiatry.* 2012;53:1167-1175.

**14.** Burke JD. An affective dimension within oppositional defiant disorder symptoms among boys: personality and psychopathology outcomes into early adulthood. *J Child Psychol Psychiatry.* 2012;53:1176-1183.

**15.** Burke JD, Hipwell AE, Loeber R. Dimensions of oppositional defiant disorder as predictors of depression and conduct disorder in preadolescent girls. *J Am Acad Child Adolesc Psychiatry.* 2010;49:484-492.

**16.** Herzhoff K, Tackett JL. Subfactors of oppositional defiant disorder: converging evidence from structural and latent class analyses [published online ahead of print Apr 2015]. *J Child Psychol Psychiatry. .* DOI: 10.1111/jcpp.12423.

**17.** Lavigne JV, Gouze KR, Bryant FB, Hopkins J. Dimensions of Oppositional Defiant Disorder in Young Children: Heterotypic Continuity with Anxiety and Depression. *J Abnorm Child Psychol.* Aug 2014;42(6):937-951.

**18.** Leadbeater BJ, Homel J. Irritable and defiant sub-dimensions of ODD: their stability and prediction of internalizing symptoms and conduct problems from adolescence to young adulthood. *J Abnorm Child Psychol.* Apr 2015;43(3):407-421.

**19.** Savage J, Verhulst B, Copeland W, Althoff RR, Lichtenstein P, Roberson-Nay R. A genetically informed study of the longitudinal relation between irritability and anxious/depressed symptoms. *J Am Acad Child Adolesc Psychiatry.* May 2015;54(5):377-384.

**20.** Stringaris A, Zavos H, Leibenluft E, Maughan B, Eley TC. Adolescent irritability: phenotypic associations and genetic links with depressed mood. *Am J Psychiatry.* Jan 2012;169(1):47-54.

**21.** Whelan YM, Leibenluft E, Stringaris A, Barker ED. Pathways from maternal depressive symptoms to adolescent depressive symptoms: the unique contribution of irritability symptoms. *J Child Psychol Psychiatry.* Oct 2015;56(10):1092-1100.

**22.** Whelan YM, Stringaris A, Maughan B, Barker ED. Developmental continuity of oppositional defiant disorder subdimensions at ages 8, 10, and 13 years and their distinct psychiatric outcomes at age 16 years. *J Am Acad Child Adolesc Psychiatry.* Sep 2013;52(9):961-969.

**23.** Deveney CM, Hommer RE, Reeves E, et al. A prospective study of severe irritability in youths: 2- and 4-year follow-up. *Depress Anxiety.* May 2015;32(5):364-372.

**24.** Stringaris A, Baroni A, Haimm C, et al. Pediatric Bipolar Disorder Versus Severe Mood Dysregulation: Risk for Manic Episodes on Follow-Up. *J Am Acad Child Adolesc Psychiatry.* Apr 2010;49(4):397-405.

**25.** Higgins JP, Thompson SG, Deeks JJ, Altman DG. Measuring inconsistency in meta-analyses. *BMJ.* Sep 6 2003;327(7414):557-560.

**26.** Egger M, Davey Smith G, Schneider M, Minder C. Bias in meta-analysis detected by a simple, graphical test. *BMJ.* Sep 13 1997;315(7109):629-634.

**27.** Sterne JA, Sutton AJ, Ioannidis JP, et al. Recommendations for examining and interpreting funnel plot asymmetry in meta-analyses of randomised controlled trials. *BMJ.* 2011;343:d4002.

**28.** Aebi M, Plattner B, Metzke CW, Bessler C, Steinhausen HC. Parent- and self-reported dimensions of oppositionality in youth: construct validity, concurrent validity, and the prediction of criminal outcomes in adulthood. *J Child Psychol Psychiatry.* Sep 2013;54(9):941-949.
